# Abdominal Compartment Syndrome Following Endovascular Aneurysm Repair for Ruptured Abdominal Aortic Aneurysm: A Challenging Case

**DOI:** 10.70352/scrj.cr.25-0231

**Published:** 2025-08-28

**Authors:** Yuki Hashimoto, Shinsuke Kikuchi, Yuya Tamaru, Tsutomu Doita, Keisuke Kamada, Naoya Kuriyama, Yuri Yoshida, Daiki Uchida, Tatsuya Shonaka, Nobuyoshi Azuma

**Affiliations:** 1 Department of Vascular Surgery, Asahikawa Medical University, Asahikawa, Hokkaido, Japan; 2 Department of Vascular Surgery, Asahikawa City Hospital, Asahikawa, Hokkaido, Japan; 3 Department of Gastrointestinal Surgery, Asahikawa Medical University, Asahikawa, Hokkaido, Japan

**Keywords:** ruptured abdominal aortic aneurysm, endovascular aneurysm repair, abdominal compartment syndrome

## Abstract

**INTRODUCTION:**

Abdominal compartment syndrome (ACS) is a serious complication that can occur after endovascular aneurysm repair (EVAR) for ruptured abdominal aortic aneurysm (rAAA). Prompt recognition and appropriate management are crucial to improve patient outcomes.

**CASE PRESENTATION:**

An octogenarian with an 11-cm rAAA underwent emergent EVAR due to cardiovascular instability. Postoperatively, the patient developed ACS, necessitating open abdominal management (OAM) due to intestinal edema and retroperitoneal hematoma. Despite multiple surgical interventions, including aneurysmorrhaphy and removal of retroperitoneal hematoma, the patient experienced prolonged difficulty in abdominal closure. The following procedures were attempted for abdominal closure: 1) Dissection of skin and subcutaneous tissues from the rectus sheath on both sides. 2) Release of the external oblique muscle from the anterior layer of the rectus by longitudinally cutting the superficial fascia. 3) Closure of both rectus sheaths with horizontal mattress sutures and negative pressure wound therapy (NPWT). On POD 20, fluid accumulation from bleeding complicated abdominal closure. However, the abdominal wall was successfully closed by achieving hemostasis and using NPWT. Despite these efforts, the patient developed multiple organ failure, including respiratory and renal failure. Sepsis ultimately led to the patient's death on the 80th POD.

**CONCLUSIONS:**

ACS following EVAR for rAAA significantly impacts patient prognosis. Specific techniques for abdominal closure, as described in this case, may help minimize the duration of OAM in challenging cases.

## Abbreviations

AAA
: abdominal aortic aneurysm
ACS
: abdominal compartment syndrome
CIA
: common iliac artery
EVAR
: endovascular aneurysm repair
IABO
: intra-aortic balloon occlusion
IAP
: intra-abdominal pressure
NBCA
: N-butyl-2-cyanoacrylate
NPWT
: negative pressure wound therapy
OAM
: open abdominal management
rAAA
: ruptured abdominal aortic aneurysm
SSI
: surgical site infection

## INTRODUCTION

rAAA is a catastrophic emergency. Emergency EVAR is recommended for its stable outcomes and improved 30-day mortality rates in unstable rAAA cases.^1)^ In unstable patients with large AAAs, ACS can develop due to retroperitoneal hematoma and intestinal edema, leading to organ perfusion abnormalities and high mortality from elevated IAP.^2,3)^ Managing tense abdominal walls or achieving closure after ACS is challenging, requiring multiple interventions to reduce intraperitoneal volume. This report describes a valuable method for abdominal closure by releasing the superficial abdominal fascia in a super-elderly patient with ACS following rAAA treated by EVAR.

## CASE PRESENTATION 

**Fig. 1:**
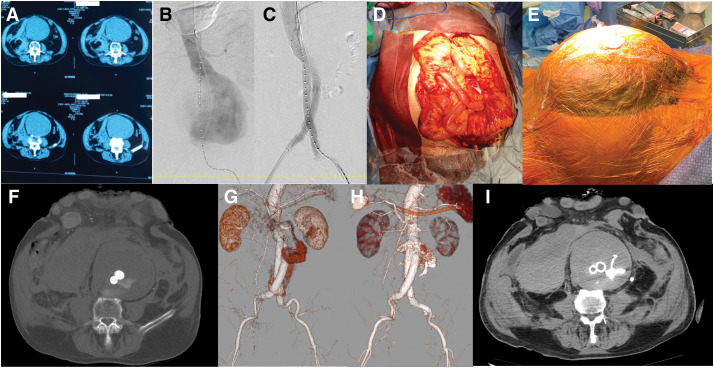
Timeline of surgical interventions. Preoperative plain CT imaging (**A**). DSA showed preoperative AAA (**B**). DSA after EVAR (**C**). Bowel findings during open abdominal decompression (**D**). Abdominal findings with Abthera and open management (**E**). Enhanced CT findings about type-1b endoleak after EVAR (**F** and **G**). Enhanced CT findings after placing iliac extender and infusion of N-butyl-2-cyanoacrylate (**H**). Retroperitoneum large hematoma and giant AAA after EVAR and additional procedure (**I**). AAA, abdominal aortic aneurysm; EVAR, endovascular aortic repair

An 89-year-old man with a history of stroke, diabetes, and lipid disorder presented with diarrhea and nausea. On examination, a giant mass was palpated in his abdomen. Non-enhanced CT revealed a giant AAA with a diameter of 11 cm. The patient was transferred to our outpatient clinic. Unfortunately, he lost consciousness due to hypotension while awaiting evaluation for the AAA. A rupture of the AAA was suspected, and CT confirmed a small retroperitoneal hematoma (**Fig. 1A**). He was immediately taken to the operating room, where his blood pressure and heart rate were 51/32 mmHg and 116 beats/min, respectively (Rutherford class III).^4)^ Under local anesthesia, the right femoral artery was punctured, and an IABO (IABO Block Balloon; Senko Medical Instrument Mfg, Tokyo, Japan) was placed through a 10 Fr sheath. Initial angiography was performed immediately after the placement of the IABO, confirming the presence of a suitable proximal neck for EVAR (**Fig. 1B**). EVAR was performed using the IABO without detailed anatomical information about the AAA from the CT film. The main body (RLT281418J) and contralateral leg (PLC141000J) stent-grafts from the GORE EXCLUDER AAA Endoprosthesis system (W.L. Gore & Associates, Newark, DE, USA) were primarily placed. Despite the short CIAs as distal sealing zones, stent-grafts were placed in both CIAs to maintain pelvic arterial perfusion (**Fig. 1C**). Post-EVAR angiography revealed a type-1b endoleak in both legs, necessitating the addition of a stent-graft to both CIAs (PLC161000J for left side and PLC161200J for right side; W.L. Gore & Associates). Following EVAR, the patient received substantial fluid and blood product resuscitation, including 3300 mL of extracellular fluid, 3640 mL of packed red blood cells, 3120 mL of fresh frozen plasma, and 250 mL of concentrated platelets (equivalent to 20 units). The patient developed abdominal induration, severe acidemia (pH 6.85), and elevated lactate levels (97 mg/dL). In our institution Asahikawa Medical University, IAP was assessed via bladder pressure measurement. The patient was placed in the supine position, and 50 mL of sterile saline was instilled into the bladder through the urinary catheter. The drainage port was clamped, and a three-way stopcock connected to a vertical pressure tube was used to measure the hydrostatic pressure from the bladder level. This method provided IAP values in cmH_2_O, which were subsequently converted to mmHg (1 mmHg ≈ 1.36 cmH_2_O) for consistency with ACS diagnostic standards. Bladder pressure measured 32 cmH_2_O (23.5 mmHg), exceeding the threshold for ACS. These findings prompted OAM due to ACS and sustained intra-abdominal hypertension (**Fig. 1D**). A 350 mL hematoma was aspirated, and no bowel ischemia was detected. The intestinal tract was wrapped in a protective layer of AbThera (3M, Saint Paul, MN, USA), stored in the abdominal cavity, and the wound was covered with a drape (**Fig. 1E**). The operation lasted 255 minutes, and the patient received 36 units (5040 mL) of red blood transfusion. Unfortunately, hypotension persisted for 6 hours post-surgery, and 1000 mL of hematogenous drainage was observed from the AbThera. Enhanced CT revealed hematoma formation due to a type-1b endoleak, necessitating reintervention (**Fig. 1F**, **1G**). The reintervention was promptly performed, and angiography confirmed the type-1b endoleak in the left leg. An iliac extender (PLL161007J; W.L. Gore & Associates) was placed in the left leg after embolization of left internal iliac artery, and NBCA infusion successfully sealed the cavity of the aneurysm (**Fig. 1H**). Postoperative CT showed a distended retroperitoneum due to a large hematoma and the giant AAA (**Fig. 1I**).

**Fig. 2:**
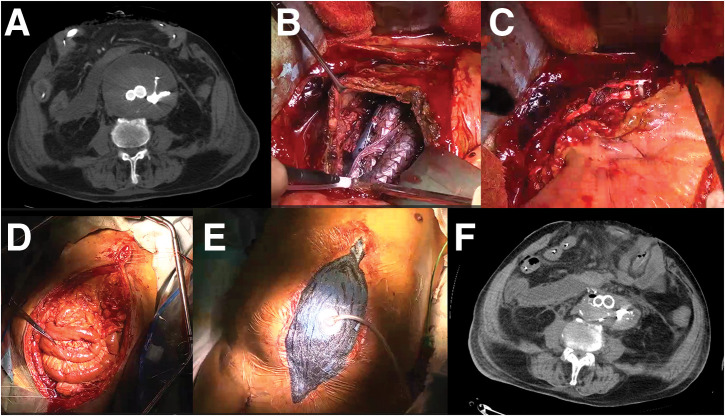
Open abdominal management after endovascular aortic repair. Reduced retroperitoneum hematoma after 2nd look procedure (**A**). Stent-graft inside an abdominal aortic aneurysm during aneurysmorrhaphy (**B**). Sutured aneurysm during aneurysmorrhaphy (**C**). Intraoperative findings showing difficulty in abdominal closure due to tissue edema (**D**). Intraoperative findings showing reapplication of negative pressure wound therapy for abdominal closure (**E**). Post-failed closure plain CT scan findings (**F**).

**Fig. 3:**
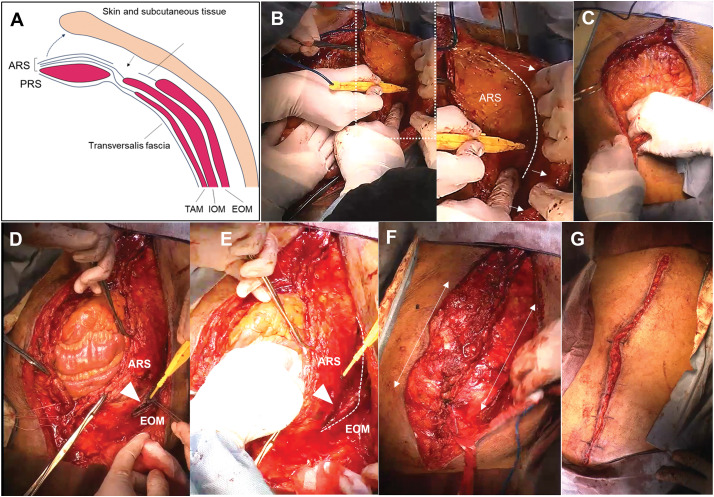
Technique of fascial release. Surgical schema of the abdominal closure technique used in abdominal closure (**A**). Intraoperative view highlighting the dissection plane between the ARS and the subcutaneous tissue; the plane is indicated by a dotted line, and the subcutaneous tissue is marked with a white arrow. (**B**). Persistent difficulty in midline closure despite completion of rectus sheath dissection (**C**). Fascial release point illustrated with an arrowhead; it marks the site of releasing the EOM from the anterior layer of the ARS (**D**). Advancement of the fascial release toward the cranial side, building upon the point shown in panel **D**; the release line is depicted with a dotted line, and the arrowhead indicates the fascial release point (**E**). Intraoperative findings of closure of both rectus sheath with horizontal mattress suture; white lines delineate the extent of bilateral fascial release (**F**). Closure of bilateral skin using horizontal mattress sutures (**G**). ARS, anterior rectus sheath; EOM, external oblique muscle; IOM, internal oblique muscle; PRS, posterior rectus sheath; TAM, transversus abdominis muscle

**Fig. 4:**
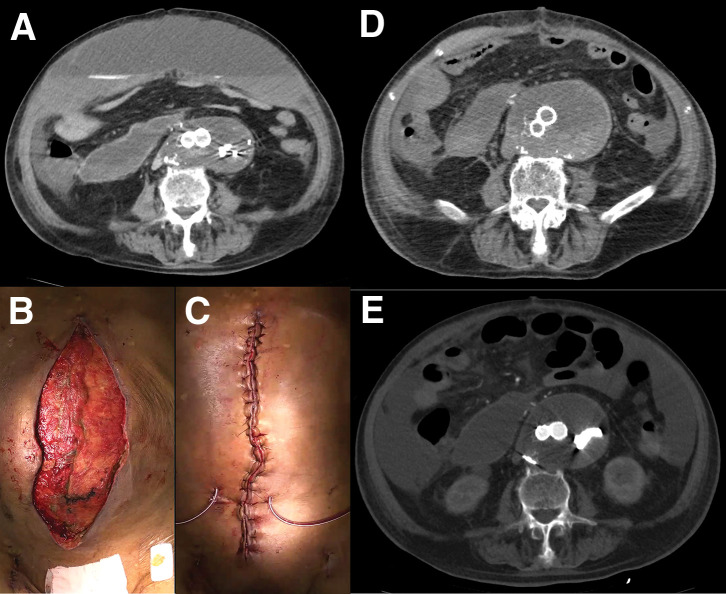
Wound closure following fascial release. Plain CT scan showing fluid accumulation between the rectus sheath and abdominal wall (**A**). Wound appearance after gauze packing, and closing abdomen (**B**, **C**). Post-closure CT scans, including a plain scan (**D**) and contrast-enhanced scan (**E**), confirmed the absence of bowel necrosis and abdominal wall hernia.

A 2nd look was performed the next day, but there was no evidence of necrosis in the intestinal tract. The retroperitoneal hematoma was removed (**Fig. 2A**). An ileus tube was inserted on POD 4 to facilitate gastrointestinal decompression and reduce intra-abdominal pressure, which was contributing to abdominal compartment physiology. This intervention also served to mitigate progressive intestinal edema and prepare for subsequent attempts at definitive closure. On POD 5, tracheostomy was performed to support long-term ventilation, given the continued need for OAM and no prospect of definitive closure. To close the abdomen, aneurysmorrhaphy was performed on POD 10 (**Fig. 2B**, **2C**). At that time, sustained gastrointestinal decompression, achieved via an ileus tube inserted on POD 4, had reduced intestinal contents and partially alleviated mesenteric congestion. In addition, bladder pressure was 16 cmH_2_O (11.8 mmHg) and the patient had achieved hemodynamic stability without catecholamines, indicating favorable conditions for closure. To further reduce intra-abdominal volume, hematoma evacuation was performed concurrently. However, despite these measures, persistent tissue edema and residual peritoneal bulk precluded primary fascial closure (**Fig. 2D**–**2F**). OAM was continued for 12 days to allow for gradual reduction of intra-abdominal pressure and resolution of visceral edema. Although preoperative assessment prior to POD 12 suggested that conventional fascial closure might be feasible, given sustained intestinal decompression, stable hemodynamics, and hematoma removal, concern remained regarding fascial tension and the risk of abdominal compartment recurrence. Therefore, fascial release technique was electively planned and performed to ensure safe and definitive closure. The negative pressure of AbThera was consistently maintained at 100 mmHg. Dressing changes were performed every 2 to 3 days, exclusively in the operating room under sterile conditions. Each application was continuously maintained until the next scheduled change. No complications directly attributable to NPWT were observed throughout the treatment course. On POD 12, the abdomen was closed by releasing the superficial fascia through the following steps (**Fig. 3A** as a surgical schema): dissection of skin and subcutaneous tissues from the rectus sheath on both sides (**Fig. 3B**), which was insufficient to close the abdomen (**Fig. 3C**); release of the external oblique muscle (EOM) from the anterior rectus sheath (ARS) by longitudinally cutting the superficial fascia (**Fig. 3D**, **3E**); and closure of both rectus sheaths with horizontal mattress sutures (**Fig. 3F**). Closure of bilateral skin was performed using horizontal mattress sutures. NPWT was applied using the Prevena Therapy system (3M), with the aim of promoting wound healing by aspirating exudate from the dissected subcutaneous plane and the separation interface between the ARS and the EOM (**Fig. 3G**). After closure on POD 12, the bladder pressure decreased to 12 cmH_2_O (8.8 mmHg) and remained low thereafter. Although primary fascial closure was achieved on POD 12, the patient subsequently developed progressive signs of systemic deterioration. On POD 15 (3 days post-closure), thrombocytopenia and anemia became evident, with platelet count declining from 75000 to 32000/μL and hemoglobin from 10.0 to 9.3 g/dL. This was accompanied by fever and gradual abdominal distention. The clinical condition worsened over the following days; by POD 20 (8 days post-closure), platelet count further decreased to 29000/μL and hemoglobin to 6.7 g/dL. Abdominal distention progressed to shock, prompting urgent CT imaging. CT revealed significant fluid accumulation between the rectus sheath and the subcutaneous tissue (**Fig. 4A**). A total of 2850 mL of fluid was aspirated, consisting of an initial 1000 mL of serous effusion followed by 1850 mL of hemorrhagic fluid. Cultures of the aspirate were negative. Despite the absence of a detectable bleeding point on the dissected surface, clinical findings were consistent with hemorrhagic shock, and bleeding was successfully controlled with cauterization and gauze packing. Contributing factors likely included coagulopathy related to disseminated intravascular coagulation (DIC), hypoalbuminemia (1.5 g/dL), and markedly reduced cholinesterase levels (82 U/L), suggestive of hepatic dysfunction. NPWT was performed for 11 days, ultimately allowing closure of the abdominal skin (**Fig. 4B**, **4C**). The patient progressed without evidence of intestinal necrosis, intra-abdominal organ ischemia, or ventral hernia during this period (**Fig. 4D**, **4E**). Despite efforts, the patient experienced multiple organ failure, including respiratory failure requiring ventilation and renal failure necessitating dialysis. Unfortunately, sepsis led to the patient’s death on the 80th POD.

## DISCUSSION

Mortality rates of octogenarians following rAAA are high, with EVAR showing superior mortality and morbidity rates compared with open surgical repair.^5)^ However, the development of ACS should always be considered in treatment following EVAR for rAAA. The incidence of ACS in EVAR for rAAA is approximately 8%. In this emergency context, the patient presented with severe hypotension and was at high cardiovascular risk, requiring urgent intervention. The initial EVAR was performed under local anesthesia using non-enhanced CT imaging, as contrast-enhanced studies were not strongly recommended due to hemodynamic instability. Consequently, anatomical assessment of the distal sealing zones, particularly the short CIAs, was limited at the time of decision-making. Given the short CIA anatomy, the risk of a type-1b endoleak was inherently high and should have been proactively addressed. Although both IIAs were preserved to maintain pelvic perfusion, this strategy may have increased the likelihood of endoleak, particularly under compromised sealing conditions. In retrospect, preemptive embolization of the IIAs and stent-graft landing in the EIAs would have offered a more secure distal seal. Such an approach might also have allowed the use of NBCA during the initial intervention, thereby mitigating hematoma progression and reducing the risk of ACS. NBCA embolization was ultimately reserved for the 2nd reintervention, once the leak source became clearly identifiable and the justification for aggressive control, based on the threat of ACS, was more evident. Furthermore, we now highlight that ACS-related complications were closely linked to endoleak persistence and EVAR failure, underscoring the importance of thorough anatomical evaluation and timely leak control in high-risk emergency settings. This case illustrates that even under emergent conditions, anticipating anatomical limitations and selecting a definitive endovascular strategy may critically influence the downstream clinical course.

ACS is defined as a sustained elevation of IAP exceeding 20 mmHg, either with an abdominal perfusion pressure below 60 mmHg or independently.^2)^ ACS can lead to multiple organ failure, making its management crucial for the prognosis of rAAA. Previous studies have identified risk factors for ACS development, including the use of IABO, transfusion of more than 3 units of blood, and prolonged activated partial thromboplastin time.^6)^ These factors contribute to increased intra-abdominal organ volume, often due to postoperative bowel edema and the formation of retroperitoneal hematoma. To facilitate early detection of ACS, IAP should ideally be monitored using a transducer-based technique, which is considered the standard method in high-resource settings due to its superior accuracy, reproducibility, and ability to provide continuous measurement in mmHg.^7)^ As recommended by the World Society of the Abdominal Compartment Syndrome (WSACS), this approach is particularly suitable for ICU and perioperative settings.^8)^ However, in the present case involving rAAA treated with EVAR, the patient required massive fluid resuscitation and transfusion, both of which are established risk factors for ACS development after endovascular repair.^9)^ Under these emergent conditions with pronounced abdominal wall tension and limited time for equipment setup, we employed the hydrostatic (water column) method for initial IAP evaluation. Although less reproducible, this method allowed for prompt screening of elevated intra-abdominal pressure and facilitated timely decompressive decision-making. Moreover, in cases requiring prolonged OAM, transducer-based monitoring may be reintroduced once the patient is stabilized. In acute settings, however, hydrostatic measurement remains a practical and sufficiently reliable option for guiding urgent clinical decisions. The current case involved hypertensive abdominal wall hematoma and required intestinal evacuation, with the risk factors. Strategic intervention is crucial to prevent intestinal necrosis and multiple organ failure during hospitalization. The primary management principle is decompression via abdominal cavity access. OAM is essential for severe abdominal conditions in ACS, but there is no universally optimal method due to the complexity of clinical scenarios. The report discusses technical tips for abdominal closure after prolonged OAM for ACS due to rAAA treated by EVAR.

There are several approaches to OAM to improve increased IAP. In this regard, a critical clinical issue to consider is the increase in infectious complications after 8 days of maintaining an open abdomen, which significantly impairs the ability to achieve primary fascial closure. The duration of OAM and the frequency of dressing changes predispose patients to infections,^10)^ underscoring the necessity of achieving abdominal closure as swiftly as physiological conditions allow. One method involves using a patch to interpose prosthetic material between the fascial margins, which is meticulously sutured into position. This approach mitigates fascial retraction and allows provisional closure of the fascial layer but is susceptible to fascial necrosis, especially when tension or iterative suturing is excessive.^11)^ In cases of fascial compromise, such as the presenting case, alternative techniques are preferred over patch closure. NPWT is another modality that applies negative pressure to the abdominal cavity.^12)^ NPWT not only reduces fascial retraction, facilitating transient closure, but also provides continuous suction for unbroken drainage of ascitic fluid. This mechanism expedites the resolution of adverse events, such as hemorrhage. In this clinical context, we implemented a sponge-based NPWT, AbThera.

Effective abdominal closure after OAM often requires various fascial release techniques to reduce tension and facilitate closure. One such technique is the external oblique muscle release, which involves making a longitudinal incision in the superficial fascia to release the external oblique muscle from the anterior layer of the rectus sheath.^13)^ This method helps reduce fascial tension and intra-abdominal pressure, making abdominal closure easier. However, SSI developed in 31% of cases who received component release ventral hernia repair, highlighting the need to pay attention to SSI as a complication.^14)^ When combined with NPWT, it enhances the effectiveness of the closure by preventing fascial contraction,^15)^ promoting continuous drainage of ascitic fluid, and aiding in the early resolution of complications such as hemorrhage. In the current case, delayed hemorrhage developed after NPWT. In patients with poor general condition and a tendency to bleed, such as those with disseminated intravascular coagulation, careful monitoring for delayed bleeding from dissection surfaces is required. The use of fascial release techniques in OAM is supported by various studies. For instance, a study by Cheatham et al. highlights the effectiveness of NPWT in managing open abdomens, emphasizing its role in preventing fascial retraction and promoting early closure.^16)^ Another study by Miller et al. discusses the benefits of external oblique muscle release in reducing intra-abdominal pressure and facilitating abdominal closure.^17)^ These techniques are crucial in managing patients with severe abdominal conditions, ensuring effective closure and reducing the risk of complications such as ventral hernias and organ ischemia.

In the present case, delayed hemorrhagic events after fascial closure were most likely multifactorial. Several systemic conditions have been implicated as contributing factors rather than technical shortcomings of the fascial release method itself. First, prolonged OAM beyond 8 days has been associated with a significantly increased risk of SSI and intra-abdominal contamination due to sustained exposure of dissected tissue surfaces and delayed fascial closure.^18)^ Second, component separation techniques and fascial dissection inherently generate wide raw surfaces, which pose risks for both infection and microvascular bleeding.^14)^ Third, hepatic dysfunction, demonstrated by hypoalbuminemia (1.5 g/dL) and markedly decreased cholinesterase levels (82 U/L), may have impaired both coagulation and innate immunity. These factors are recognized contributors to bacteremia and bleeding tendency in cirrhotic or septic patients.^18)^ Fourth, transient DIC likely exacerbated systemic coagulopathy and microvascular leakage. DIC is known to significantly elevate mortality among surgical patients with systemic inflammation or sepsis.^19)^ These risk factors were collectively considered during postoperative management and are now discussed in detail to clarify the complexity of the bleeding events. We believe that these systemic conditions played a predominant role in the delayed hemorrhage, rather than technical aspects of the fascial release itself. Furthermore, the high susceptibility to infection following fascial release techniques highlights the critical importance of implementing rigorous infection control measures, particularly in patients with compromised immunity or those undergoing prolonged OAM.

In summary, emergency EVAR was performed on a patient with rAAA. OAM effectively mitigated the risk of ACS and prevented abdominal organ ischemia. Subsequent stepwise abdominal closure with NPWT allowed for successful closure without the development of a ventral hernia.

## CONCLUSIONS

ACS following EVAR for rAAA significantly impacts patient prognosis. Specific techniques for abdominal closure, such as the component release method, may help minimize the duration of OAM in challenging cases.

## DECLARATIONS

### Availability of data and materials

The data used for this case report are available from the corresponding author upon request.
